# KIR-HLA footprints and NK cell-mediated recognition of HIV-1

**DOI:** 10.1186/1742-4690-9-S2-P170

**Published:** 2012-09-13

**Authors:** CF Thobakgale, J Carlson, J Wright, N van Teijlingen, M Jaggernath, P Goulder, BD Walker, M Carrington, D Heckerman, M Altfeld, T Ndung'u

**Affiliations:** 1 University of KwaZulu-Natal, Durban, South Africa; 2 Microsoft Research, Redmond, WA, USA; 3 Ragon Institute of MIT, MGH and Harvard, Boston, MA, USA; 4 University of Oxford, Oxford, UK; 5 Cancer and Inflammation Program, Laboratory of Experimental Immunology, Frederick, MD, USA

## Background

Increasing data suggest an important role of KIR+ NK cells in the control of HIV-1, however the precise mechanisms on how NK cells recognize HIV-1-infected cells remain poorly understood. KIRs can bind to HLA class I molecules, but the binding affinity of these interactions is dependent on the HLA class I presented epitope. Recent reports have suggested that sequence variations within HIV-1 epitopes presented by HLA class I can affect the binding of inhibitory KIRs expressed on NK cells, potentially modulating NK cell responses to infected cells. Here we investigated whether HIV-1 might adapt to the combined KIR/HLA genotypes on a population level to identify areas within HIV-1 that might be targeted by KIR+ NK cells.

## Methods

HIV-1 Gag was sequenced in 390 untreated chronically clade C infected individuals from KwaZulu-Natal, South Africa. All study subjects were HLA class I and KIR typed. Phylogenetically-corrected logistic regression analysis of KIR/HLA associated Gag sequence polymorphisms was performed and q-values were used for multiple test correction.

## Results

A total of 93 sequence polymorphisms significantly associated with the combined HLA/KIR genotypes were identified (p<0.05), 6 of them with a false-positive rate of less than 20% (q<0.2). These significant associations were independent of previously identified KIR or HLA-linked polymorphisms.

## Conclusion

This study identified several sequence polymorphisms within HIV-1 Gag that were significantly associated with the expression of combined KIR/HLA genotypes at the population level, indicating adaptation of HIV-1 to NK cell mediated immune pressure. KIR/HLA class I binding studies in the context of the sequence polymorphisms and studies for NK cell function are ongoing to determine the consequence of these sequence changes for NK cell-mediated recognition of HIV-1-infected cells.

